# Clinical Findings, Treatment and Long-Term Outcome in 37 Cats with Non-Associative Immune-Mediated Hemolytic Anemia: A Retrospective Observational Study of Two Treatment Cohorts

**DOI:** 10.3390/ani16172727

**Published:** 2026-09-02

**Authors:** Lisa-Maria Kulmer, Pavlos G. Doulidis, Iwan A. Burgener, Nicole Luckschander-Zeller

**Affiliations:** Clinical Unit of Small Animals Internal Medicine, Department for Companion Animals and Horses, University of Veterinary Medicine Vienna, 1210 Vienna, Austria

**Keywords:** IMHA, cyclosporin A, glucocorticoids, prednisolone, hemolytic anemia, cats

## Abstract

**Background:** Non-associative immune-mediated hemolytic anemia (IMHA) is uncommon in cats and carries substantial mortality. Glucocorticoids are first-line treatment, and cyclosporin A (CsA) is frequently added, but data on the clinical course and long-term outcome are limited. We describe clinical findings, treatment, adverse events and outcome in cats with non-associative IMHA. **Methods:** In this retrospective observational single-center study (October 2014–October 2024), 37 cats diagnosed by the 2019 ACVIM consensus criteria were included; 15 received prednisolone alone (PG) and 22 prednisolone combined with CsA (CPG), allocated at the clinician’s discretion. Between-cohort comparisons are exploratory. **Results:** Cats in the CPG cohort presented with more severe anemia. Overall mortality was 24.3%, with all deaths within four weeks; survival to discharge was 13/15 (PG) and 19/22 (CPG). Median follow-up was 50 [8–430] and 70 days [12–330]. Two cats achieved sustained treatment-free remission of 4.2 and 1.9 years. Five cats relapsed, four while receiving ongoing immunosuppression. **Conclusions:** Most cats survived the acute phase but required prolonged immunosuppression, and sustained treatment-free remission was uncommon. Relapses occurred predominantly during ongoing therapy rather than after discontinuation. Because CsA was used preferentially in more severely affected cats, comparative efficacy cannot be assessed and remains to be addressed prospectively.

## Simple Summary

Immune-mediated hemolytic anemia is a disease in which the immune system attacks and destroys the body’s own red blood cells, leaving affected animals severely anemic. It is less common in cats than in dogs. The standard treatment is prednisolone, a glucocorticoid that suppresses the immune system, and cyclosporin A is often added to strengthen this effect and to allow the prednisolone dose to be reduced. We reviewed the records of 37 cats treated at a single hospital to describe how the disease presented, how it was treated and how the cats fared over time. Some cats received prednisolone alone and others the combination; because the sicker cats were the ones given cyclosporin, the two treatments cannot be fairly compared, and we therefore describe both groups rather than judging which treatment works better. Most cats survived the first weeks of illness, but the majority needed medication long term, and only two cats remained healthy for years without any treatment. When the disease returned, it usually did so while the cats were still receiving medication. Prospective studies are needed to determine whether adding cyclosporin benefits cats with this disease.

## 1. Introduction

Immune-mediated hemolytic anemia (IMHA) is a rare but potentially life-threatening condition in cats, characterized by immune-mediated destruction of red blood cells [1,2,3]. When no underlying disease or triggering factor associated with antibody production can be identified, IMHA is classified as non-associative [1,2]. Non-associative IMHA requires prompt immunosuppressive treatment and supportive care [3]. No breed or sex predisposition has been described in cats, although one study reported an overrepresentation of male cats [2]. Across published studies, cats with non-associative IMHA are generally diagnosed at a young age, with reported ages most frequently ranging between 2 and 6 years [2,3].

According to the American College of Veterinary Internal Medicine (ACVIM) consensus statement 2019, the diagnosis of IMHA requires evidence of immune-mediated red blood cell destruction (saline autoagglutination, spherocytosis, or a positive direct antiglobulin test) together with at least one marker of hemolysis, such as hyperbilirubinemia, bilirubinuria, hemoglobinemia, or hemoglobinuria [1]. In cats, the identification of spherocytes is difficult because feline erythrocytes are small and lack central pallor [2].

Classification as non-associative IMHA requires the exclusion of potential underlying diseases and triggering factors, such as infection with hemotropic mycoplasmas or feline leukemia virus (FeLV), neoplasia, drug exposure, and intoxication. Evidence supporting vaccination as a trigger remains limited [1,2,4].

Immunosuppressive agents remain the cornerstone of treatment, with glucocorticoids such as prednisolone representing the first-line choice. Although cats are generally considered less susceptible to glucocorticoid-associated adverse effects than other species, congestive heart failure, diarrhea, and iatrogenic hyperadrenocorticism have been reported [5,6]. Glucocorticoid treatment has also been identified as a risk factor for diabetes mellitus in cats [7].

Cyclosporin A (CsA) is a calcineurin inhibitor that suppresses T-cell activation and proliferation by inhibiting interleukin-2 transcription, thereby targeting a pathway distinct from the predominantly phagocyte-directed effects of glucocorticoids [8]. It is therefore increasingly incorporated into feline treatment protocols, both to enhance immunosuppression and for its potential glucocorticoid-sparing effect, despite a lack of evidence supporting its efficacy in feline IMHA [4]. The most common adverse effects of oral CsA are mild gastrointestinal signs, which are usually transient, dose-dependent, and manageable with supportive treatment, and therefore rarely require drug discontinuation [7]. Other reported adverse effects include gingival hyperplasia, polyphagia, polydipsia, hyperactivity, and hypertrichosis. More serious but infrequent complications, such as acute bullous keratopathy, hemolytic uremic syndrome, or severe toxicity, have been reported only sporadically [7,8].

Reported IMHA-associated mortality in cats is approximately 23.5–30% [2,9], whereas rates of approximately 30–52% have been reported in dogs [10,11,12]. Thromboembolic complications are common in dogs with IMHA but have only been reported sporadically in cats [13,14]. In cats, IMHA is classified as a low-risk condition for thrombosis, and antithrombotic therapy is recommended only in the presence of additional risk factors such as cardiomyopathy, heartworm disease, protein-losing enteropathy or nephropathy, sepsis or hyperadrenocorticism, or when more than one risk factor coexists [13].

Data on the clinical course and outcome of non-associative IMHA in cats remain limited. The available feline literature consists of small case series in which treatment protocols were heterogeneous, cats receiving combination therapy were not analyzed separately, and long-term follow-up was rarely reported [2,3,4,9]. In particular, little is known about how frequently cats achieve sustained remission after discontinuation of immunosuppressive therapy and how often relapses occur during ongoing treatment.

The aim of this retrospective observational study was to describe the clinical and clinicopathological findings at presentation, the treatment protocols used, adverse events, and the short- and long-term outcome in cats with non-associative IMHA managed at a single referral center, and to report these findings separately for cats treated with prednisolone alone (PG) and cats treated with prednisolone combined with CsA (CPG). Because treatment was allocated at the clinician’s discretion rather than randomly, no comparison of treatment efficacy between the two cohorts was intended or attempted; between-cohort comparisons are reported descriptively to characterize the two populations.

## 2. Materials and Methods

### 2.1. Study Population

In this retrospective observational study, cases were identified by a search of the electronic medical record system (Tierspitalinformationssystem, TIS) of the University Clinic for Small Animals, Vetmeduni Vienna, Austria, between October 2014 and October 2024, using the keywords “immune-mediated hemolytic anemia”, “IMHA”, “Coombs test” and “feline”. Cats were included if they fulfilled all of the following criteria, in accordance with the 2019 ACVIM Consensus Statement: (1) anemia (hematocrit or packed cell volume < 27%); (2) evidence of immune-mediated erythrocyte destruction, defined as a positive direct antiglobulin test (DAT) and/or a positive saline agglutination test; and (3) at least one marker of hemolysis (hyperbilirubinemia, hemoglobinemia, or hemoglobinuria). Spherocytosis was not used as an inclusion criterion, owing to the recognized difficulty of identifying spherocytes in cats. The minimum diagnostic investigation required to classify a case as non-associative IMHA comprised exclusion of recent toxin or drug exposure; qualitative testing for feline leukemia virus (FeLV) antigen and feline immunodeficiency virus (FIV) antibody (SNAP FIV/FeLV Combo Plus Test, IDEXX Laboratories, Westbrook, ME, USA); and serum biochemistry, abdominal ultrasonography, thoracic radiography and PCR testing for *Mycoplasma haemofelis*, *Candidatus* Mycoplasma haemominutum and *Candidatus* Mycoplasma turicensis. FeLV infection was excluded by antigen testing only; provirus PCR was not performed in any cat. Cats in which CsA was introduced more than four weeks after initiation of prednisolone were excluded a priori, as such cases could not be meaningfully assigned to either treatment cohort.

### 2.2. Data Collection

For each cat, the following data were recorded from the medical records: signalment (breed, sex, neuter status, age), body temperature at presentation, blood type, and vaccination status, including whether vaccination had been administered within 30 days before the onset of clinical signs.

Laboratory data were recorded at presentation, during the initial hospitalization and, where available, at follow-up. Complete blood counts were performed using an ADVIA 2120i hematology analyzer (Siemens Healthcare Diagnostics GmbH, Vienna, Austria) and serum biochemistry using a Cobas c 501 analyzer (Roche Diagnostics, Vienna, Austria). At presentation, the hematocrit (HCT), hemoglobin concentration, total reticulocyte count, platelet count, total bilirubin, alanine aminotransferase (ALT), alkaline phosphatase (ALKP), creatinine, urea and serum amyloid A were recorded. During the initial hospitalization, the lowest HCT, the highest and lowest leukocyte and neutrophil counts, and the lowest lymphocyte count were recorded, together with the number of blood transfusions administered. At follow-up, the HCT on days 14 and 30 after initiation of treatment and the time to HCT normalization (≥27%) were recorded. A direct antiglobulin (Coombs) test (direct method, microtiter plate) and a saline agglutination test were performed in all cats. The presence of spherocytes was noted descriptively. All blood smears were reviewed by a board-certified clinical pathologist. Prednisolone was initiated at an immunosuppressive dosage and subsequently tapered guided by clinical and hematologic response, by approximately 25% every 2–4 weeks once the HCT had stabilized. Cyclosporin A was used as an adjunctive agent, added at the discretion of the attending clinician. The initial CsA dosage and the interval from treatment initiation to CsA introduction were recorded. Therapeutic drug monitoring of CsA was not performed in any cat. The number of blood transfusions administered during the initial hospitalization was recorded. Transfusion was given at the clinician’s discretion, based on a packed cell volume below 20% and/or clinical signs of decompensation related to anemia (e.g., tachypnea, tachycardia).

Adverse events during treatment were extracted retrospectively from the medical records and comprised polyphagia, polyuria/polydipsia, acute kidney injury, hyporexia/inappetence, bacterial cystitis, excessive hair loss, lethargy, vomiting/nausea, feline upper respiratory tract disease, congestive heart failure, diabetes mellitus, pica and diarrhea.

### 2.3. Outcome

Follow-up information was obtained from the medical records and, where necessary, by telephone contact with the referring veterinarian or owner. The follow-up period was defined as the interval from initiation of treatment to the last documented contact. The number of cats with data available is reported at each time point.

Survival to discharge from the initial hospitalization was recorded; cats discharged against veterinary advice were counted as having survived to discharge. Outcome was classified at the last documented contact as complete remission, stable disease, or death/euthanasia. Complete remission was defined as a hematocrit within the reference interval that remained stable for at least three months after complete discontinuation of all immunosuppressive medication. Stable disease was defined as a packed cell volume maintained ≥27% under ongoing immunosuppressive therapy, without recurrence of hemolysis or the need for further transfusion. Relapse was defined as recurrence of anemia with evidence of hemolysis after initial stabilization. For each relapse it was recorded whether it occurred during ongoing immunosuppressive therapy or after complete discontinuation. For each death, it was recorded whether euthanasia or death was attributable to IMHA or its treatment. Mortality was assessed at 30 and 90 days.

### 2.4. Statistical Analysis

All statistical analyses were performed using GraphPad Prism version 11.0.0 for Windows (GraphPad Software, Boston, MA, USA). Continuous variables are reported as median and interquartile range (IQR) and were compared between groups using the Mann–Whitney U test. Categorical variables are reported as counts and proportions and were compared using Fisher’s exact test. Significance was determined by *p* < 0.05 for all comparisons. Because treatment allocation was not randomized, all between-group comparisons are reported as descriptive characterizations of the two cohorts and not as tests of treatment efficacy. Where a between-group difference is reported, an effect size with a 95% confidence interval is given.

## 3. Results

### 3.1. Study Population

Thirty-eight cats met the inclusion criteria during the study period (October 2014–October 2024). One cat, in which CsA was initiated only on day 1155, was excluded a priori, leaving 37 cats for analysis: 15 received prednisolone alone (PG) and 22 received prednisolone combined with cyclosporin A (CPG).

In the PG group, 10 cats were male and 5 were female, with a median age of 4 years [1.0–9.0] and a median body temperature at presentation of 38.4 °C [38.0–39.2]. Thirteen cats were Domestic Shorthair and 2 were Maine Coon. In the CPG group, 8 cats were male and 14 were female, with a median age of 5 years [2.0–8.5] and a median body temperature of 38.0 °C [37.9–38.5]. Nineteen were Domestic Shorthair, 2 British Shorthair and 1 Maine Coon. All cats were neutered. The sex distribution did not differ between groups (*p* = 0.10).

All 37 cats were blood type A. Vaccination (RCP) had been recorded in 9/15 PG and 12/22 CPG cats, with no difference between groups (*p* > 0.99). In two cats (one per group), vaccination had been administered approximately two weeks before the onset of clinical signs. No other potential trigger was identified in either cat, so both were retained in the non-associative cohort. A direct antiglobulin (Coombs) test and a saline autoagglutination test were performed in all 37 cats. The Coombs test was positive in 14/15 (93%) PG and 19/22 (86%) CPG cats, and saline autoagglutination was positive in 3/15 (20%) PG and 8/22 (36%) CPG cats. Four cats (1 PG, 3 CPG) had a negative Coombs test and were included on the basis of a positive saline autoagglutination test. All cats tested negative for FIV and FeLV (point-of-care ELISA) and for hemotropic mycoplasmas by PCR.

### 3.2. Treatment Protocols

The initial prednisolone dosage was a median of 4 mg/kg/day in both groups (PG [3.0–4.0]; CPG [4.0–4.0] mg/kg/day). Prednisolone was administered twice daily in 31/37 cats and once daily in 6/37. No standardized dosing protocol was applied across the study period. In the CPG group, CsA was initiated a median of 1.5 days after starting prednisolone [1,2,3,4]. All included cats began CsA within the first 9 days of treatment. The initial CsA dosage was a median of 10 mg/kg/day [5–10 mg/kg/day], administered twice daily in 14/22 cats and once daily in 8/22.

### 3.3. Hematological and Biochemical Parameters

**Figure 1:**
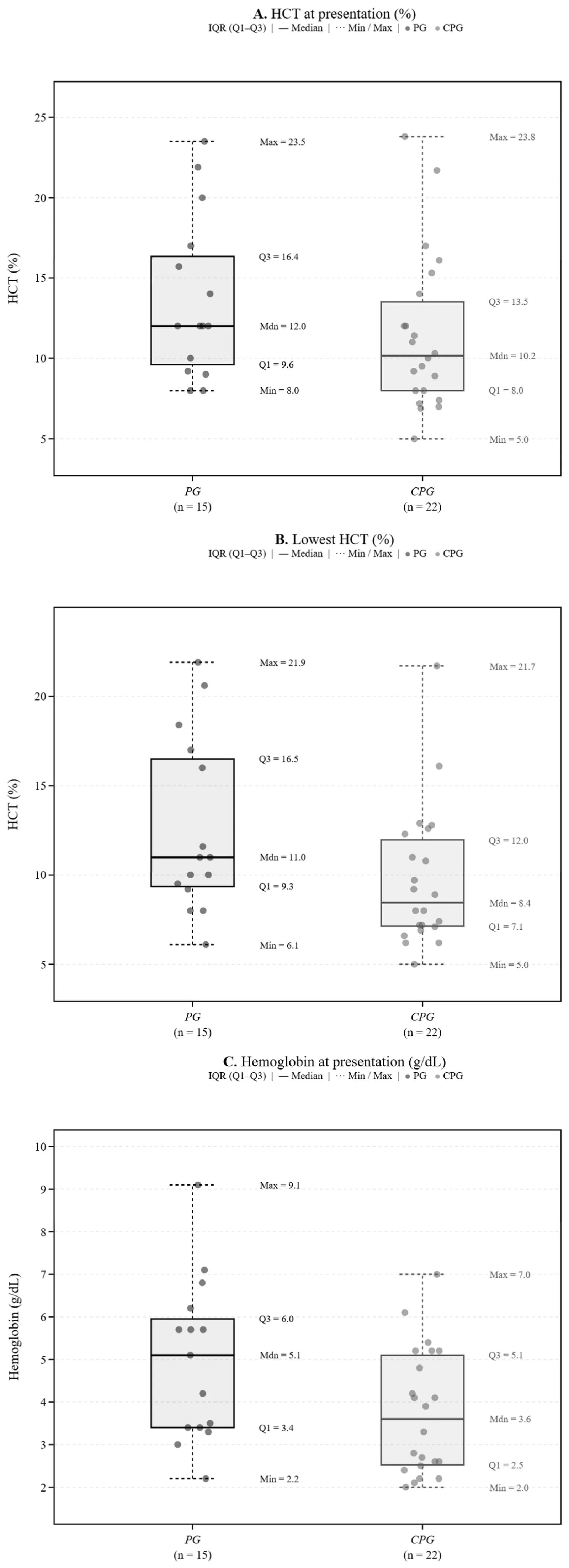

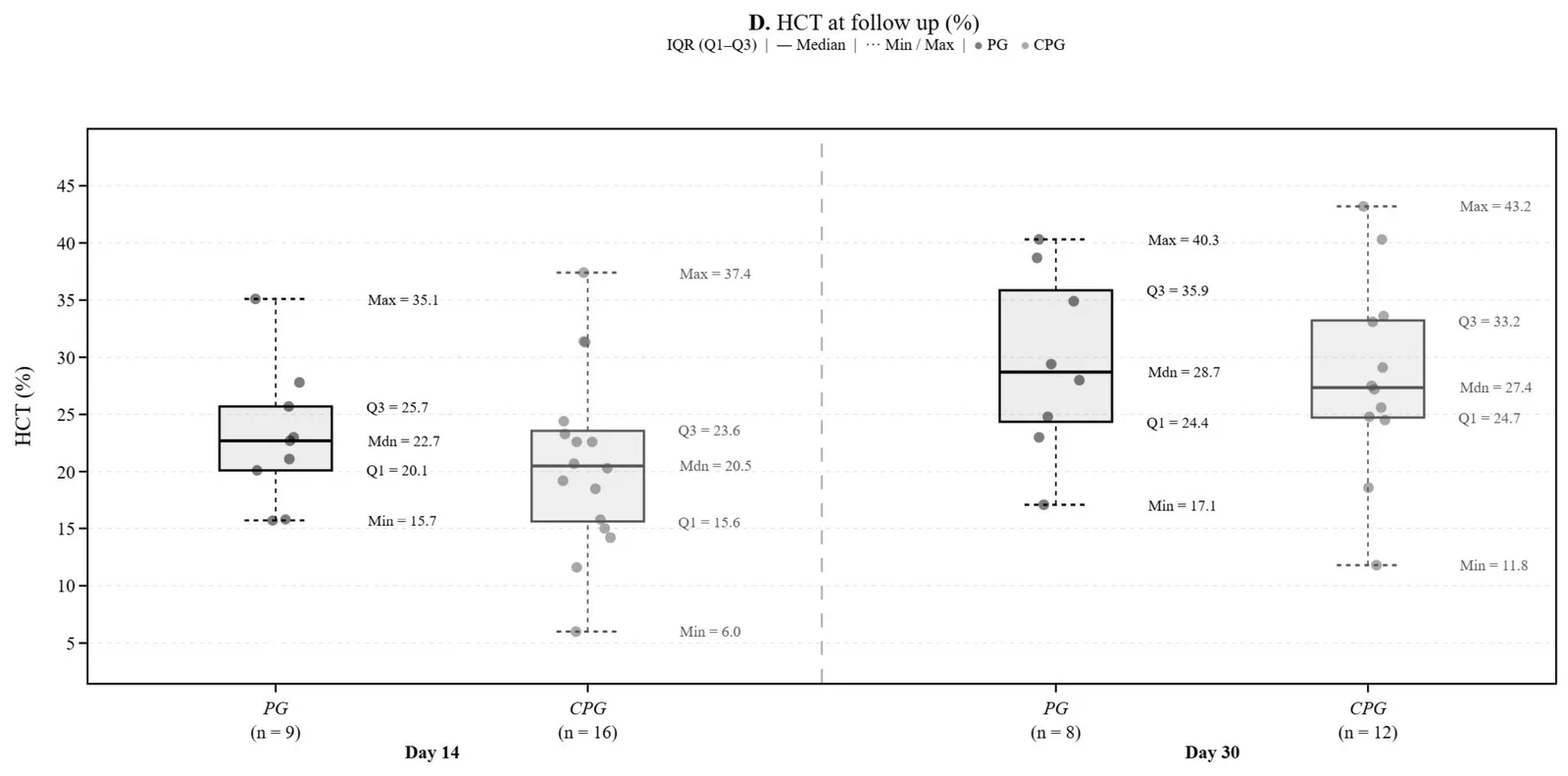
Hematocrit and hemoglobin in cats with non-associative immune-mediated hemolytic anemia treated with prednisolone alone (PG) or with prednisolone combined with cyclosporin A (CPG). (**A**) Hematocrit at presentation; (**B**) lowest hematocrit during the initial hospitalization; (**C**) hemoglobin at presentation; (**D**) hematocrit at follow-up on day 14 and day 30.

Hematological and biochemical variables at presentation and during the initial hospitalization are summarized in Table 1. Hematocrit and hemoglobin values are also shown in Figure 1. The only variable that differed nominally between cohorts was hemoglobin at presentation, which was lower in the CPG cohort (PG 5.1 [3.4–6.2] g/dL; CPG 3.6 [2.5–5.2] g/dL; *p* = 0.03; Figure 1C). All other parameters did not differ. HCT at presentation (PG 12.0% [9.2–17.0]; CPG 10.2% [7.9–14.3]; *p* = 0.12; Figure 1A) and the lowest HCT during the initial hospitalization (PG 11.0% [9.4–16.5]; CPG 8.5% [7.1–12.4]; *p* = 0.06; Figure 1B) did not differ, although the nadir was lower in the CPG group. HCT on day 14 (*p* = 0.47), day 30 (*p* = 0.75) did not differ. Hematocrit normalized (≥27%) in 9/15 (60%) PG and 10/22 (45%) CPG cats, after a median of 22 and 34.5 days, respectively (*p* = 0.84). Six PG and 12 CPG cats never reached a hematocrit of ≥27% during the observation period. Time to normalization therefore describes only those cats that recovered and does not account for cats that died or failed to normalize. Reticulocyte counts did not differ between cohorts (*p* = 0.57), and only 4/15 PG and 6/22 CPG cats had an absolute reticulocyte count above the laboratory threshold of 60 × 10^3^/µL. Hematocrit on days 14 and 30 is shown in Figure 1D. The lowest white blood cell count did not differ between groups (PG 7182 [5060–9240]/µL; CPG 6035 [4223–7686]/µL; *p* = 0.19). Highest WBC, neutrophil counts (lowest and highest) and lowest lymphocyte count likewise did not differ (Table 1). The most severe neutropenia (57/µL) was recorded in a PG cat (lowest CPG value 369/µL). Serum biochemistry did not differ between groups: total bilirubin (*p* = 0.72), ALT (*p* = 0.69), ALKP (*p* = 0.68), urea (*p* = 0.29), creatinine (*p* = 0.80) and serum amyloid A (*p* = 0.09) were comparable.

### 3.4. Transfusion Requirement

The number of blood transfusions did not differ between groups (PG median 1 [0–1]; CPG median 1 [1–2]; *p* = 0.08). No cat received more than two transfusions. More than one transfusion was required in 1/15 (6.7%) PG and 7/22 (31.8%) CPG cats (*p* = 0.11).

### 3.5. Adverse Events

Adverse events recorded during treatment are summarized in Table 2. The most frequently recorded events were hyporexia/inappetence (3/15) in the PG group, and polyphagia and lethargy (5/22 each) in the CPG group. The proportion of cats experiencing at least one adverse event did not differ between groups (PG 8/15, 53%; CPG 14/22, 64%; *p* = 0.73) and the number of adverse events per cat was identical (both median 1 [0–3]; *p* = 0.87). Feline upper respiratory tract disease (FURTD) occurred in three CPG cats during immunosuppressive treatment. A PCR panel for feline herpesvirus type 1 and feline calicivirus was performed in all three; all were positive for FHV-1 and one was additionally positive for FCV.

### 3.6. Outcome

Median follow-up was 50 days [8–430] in the PG cohort and 70 days [12–330] in the CPG cohort (*p* = 0.70), with a range of 3–2340 and 4–1924 days, respectively. Follow-up extended beyond 30 days in 8/15 PG and 13/22 CPG cats, and beyond 90 days in 6/15 and 10/22 cats, respectively.

Thirteen of 15 (86.7%) PG and 19/22 (86.4%) CPG cats survived to discharge from the initial hospitalization. Outcome at the last documented contact was classified as complete remission, stable disease, or death/euthanasia (Table 3). Complete remission was achieved in 1/15 (6.7%) PG and 1/22 (4.5%) CPG cats, and stable disease in 10/15 (66.7%) and 16/22 (72.7%), respectively. Overall mortality was 24.3% (9/37), with no difference between cohorts (PG 4/15, 26.7%; CPG 5/22, 22.7%; *p* > 0.99). All non-survivors were euthanized within the first four weeks of treatment, and all euthanasia were attributable to IMHA or its treatment. Thirty-day and 90-day mortality were therefore identical to overall mortality in each cohort. The two cats achieving complete remission discontinued all immunosuppressive medication 820 and 649 days after initiation of treatment and remained free of relapse for a further 4.2 and 1.9 years, respectively; neither had relapsed at the last documented contact. Five cats experienced recurrence of hemolytic disease during follow-up: one in the PG and four in the CPG cohort. Four of these five relapses occurred during ongoing immunosuppressive therapy, at days 159, 184, 557 and 1875, whereas the single PG cat relapsed on day 96, twenty days after complete discontinuation of prednisolone. The cat relapsing on day 1875 presented with Evans’ syndrome and was euthanized 49 days later.

## 4. Discussion

In this retrospective observational study of 37 cats with non-associative IMHA, findings are reported separately for cats treated with prednisolone alone (PG) and cats treated with prednisolone combined with cyclosporin A (CPG). Cats in the CPG cohort presented with a lower hemoglobin concentration (*p* = 0.03), a lower hematocrit nadir (*p* = 0.06), a greater transfusion requirement (*p* = 0.08) and a higher frequency of adverse events (64% vs. 53%), all consistent with greater disease severity at presentation. This pattern reflects the preferential selection of more severely affected cats for combination therapy.

Previous studies in cats with IMHA have not identified a clear sex predisposition, although Kohn et al. noted a slight male overrepresentation (11/19) [2]. In our cohort, the overall sex distribution was approximately equal (18/37, 49% male). Between groups, however, males were proportionally more common in the PG group (10/15, 67%) than in the CPG group (8/22, 36%; *p* = 0.10), although this did not reach significance. As in previous reports, no clear breed predisposition was detected: most cats were Domestic Shorthair (86%), with 8% Maine Coon and 5% British Shorthair [2,3]. The median age at presentation was 4 years in the PG and 5 years in the CPG, which is consistent with previous studies reporting a median age of 2–6 years for cats with IMHA [3]. Type A is the most common feline blood type worldwide, reported in 72–86% of cats depending on geographic region [15,16]. Consistent with this, all cats in our study were blood type A.

RCP vaccination was documented in only 9/15 (60%) PG and 12/22 (55%) CPG cats, with no difference between groups. This relatively low recorded coverage is consistent with the declining owner compliance with preventive vaccination reported broadly in small-animal practice since the COVID-19 pandemic, although in a retrospective setting incomplete documentation cannot be excluded as a contributing factor [17]. In two cats (one per group), vaccination had been administered approximately two weeks before the onset of clinical signs. Vaccine-associated IMHA has been proposed in dogs, in which onset within roughly one month of vaccination has been described but a comparable association has not been established in cats [1,18]. Because vaccine-associated IMHA is not an established entity in cats and vaccination is extremely common, both cats were retained in the non-associative cohort, although a contributory role of vaccination can neither be confirmed nor excluded [1].

Hemoglobin at presentation was lower in the CPG group (3.6 vs. 5.1 g/dL; *p* = 0.03), and the hematocrit nadir was lower as well (8.5% vs. 11.0%; *p* = 0.06). Presenting hematocrits were low in both groups (10–12%), in keeping with previous reports of a median HCT of approximately 12% in feline IMHA [2,3]. Because early signs such as lethargy and inappetence are nonspecific, and because cats tolerate lower hematocrits than dogs, affected cats are frequently presented only at an advanced stage [2,3]. Reticulocyte counts remained below the laboratory threshold of 60 × 10^3^/µL in 11/15 PG and 16/22 CPG cats, indicating an inadequate regenerative response in the majority of cats in both cohorts. This is broadly consistent with, and in our cohort more pronounced than, the 11/19 (58%) non-regenerative cats reported by Kohn et al. [2]. It may reflect delayed reticulocytosis, evaluation at an early stage of the regenerative response, or the difficulty of recognizing early regeneration in cats [2]. Idogs, more profound anemia has been associated with greater disease severity, although not consistently as an independent prognostic factor [19]. In humans with IMHA, by contrast, profound anemia (hemoglobin < 6–8 g/dL) has consistently been associated with higher mortality, higher relapse rates and worse overall outcome [20].

Nineteen between-group comparisons were performed in Table 1, and the single significant result would not retain significance after adjustment for multiple testing (Bonferroni-adjusted *p* = 0.57; Benjamini–Hochberg *q* = 0.38). The lower hemoglobin concentration in the CPG cohort should therefore not be regarded as a robust isolated finding. The absolute difference was modest (Hodges–Lehmann median difference 1.0 g/dL, 95% CI 0.1 to 2.4 g/dL). Hematocrit at presentation and hematocrit nadir differed in the same direction but did not reach significance (2.0%, 95% CI −0.5 to 5.0, *p* = 0.13; and 2.5%, 95% CI 0.0 to 4.8, *p* = 0.06, respectively), and all three confidence intervals are compatible with differences of little clinical relevance. Taken together, these three measures of the severity of anemia are concordant in direction but imprecisely estimated. The lower neutrophil counts in the CPG cohort do not necessarily form part of the same pattern. Serum amyloid A was numerically higher in the PG cohort, so a uniformly greater inflammatory response in CPG cats is not supported by these data. The observation that cats in the CPG cohort were more severely affected therefore rests less on any individual comparison than on the fact that CsA was added at the clinician’s discretion in cats considered more severely affected.

White blood cell, neutrophil and lymphocyte counts did not differ between cohorts. The lowest neutrophil count was lower in the CPG cohort (3230 vs. 5004/µL; *p* = 0.06). Mild leukopenia, lymphopenia and neutropenia, usually still within reference limits, have been described in cats receiving CsA, so an additive immunosuppressive effect of the combination is plausible but cannot be established from these data [8]. The most profound neutropenia (57/µL) occurred in a PG cat. Lowest lymphocyte counts did not differ between groups (*p* = 0.19). This contrasts with the lymphocytosis reported in up to 32% of cats with non-associative IMHA, possibly reflecting the advanced disease stage at presentation in our cohort and the predominantly lymphopenic effect of glucocorticoid therapy [2].

ALT activity was mildly increased in both groups, consistent with hepatocellular hypoxia secondary to severe anemia, as previously reported in cats with IMHA [2,3]. Alkaline phosphatase activity remained within or close to the upper reference limit. Total bilirubin was mildly increased in both groups, in keeping with ongoing hemolysis; its relationship with outcome was not assessed in this study. Serum amyloid A was mildly increased in both groups, with higher values in the PG group, although the difference was not significant (*p* = 0.09) and measurements were available in only 9 and 12 cats, respectively. To our knowledge, SAA has not previously been reported in feline IMHA, and the following considerations are therefore exploratory. In dogs with IMHA, increased CRP indicates that hemolytic crises are accompanied by systemic inflammation [21], and accessible hematological surrogates such as the neutrophil-to-lymphocyte ratio have more recently been investigated as prognostic markers in this species [11]. As acute phase proteins are sensitive but nonspecific markers of systemic inflammatory processes, the SAA values observed here may reflect the inflammatory component of hemolysis, although no conclusion regarding diagnostic or prognostic relevance can be drawn from this sample size.

In this cohort, the addition of CsA was not associated with a measurable clinical advantage. The study design, however, does not permit this to be interpreted as an absence of benefit. Transfusion requirement did not differ (both median 1; *p* = 0.08), although repeat transfusions were numerically more frequent in the CPG group, and the time to hematocrit normalization was not shorter in the CPG group (median 34.5 vs. 22.0 days; *p* = 0.84), although this comparison includes only the 9/15 PG and 10/22 CPG cats that reached a hematocrit of ≥27%. For comparison, Kohn et al. reported that 15/17 cats reached an HCT > 25% within 8–42 days on immunosuppressive prednisolone alone [2]. Because CsA was preferentially added in cats perceived to be more severely affected, these comparisons are confounded by indication. Our data are equally compatible with the possibility that CsA enabled more severely affected cats to achieve outcomes comparable to those of less severely affected cats receiving prednisolone alone, and they therefore neither demonstrate nor exclude a clinical benefit of combination therapy. While an ACVIM consensus statement on the treatment of IMHA in dogs is available, no such guideline currently exists for cats, underscoring the need for further research in this species [22].

Adverse events were comparable between groups (PG 53%, CPG 64%; *p* = 0.73; median one per cat), with polyphagia, lethargy and transient gastrointestinal signs predominating, as expected for glucocorticoids and CsA. Gastrointestinal signs are the most common and usually transient adverse effects of CsA in cats [6]. The numerically higher frequency in the CPG group was not significant and is compatible with the greater disease severity of these cats. Lethargy most likely reflected the severe anemia in these cats; it has, however, also been reported as an uncommon adverse effect of systemic glucocorticoids in dogs [23]. All three cats with upper respiratory tract disease tested positive for FHV-1, and two of the three had documented RCP vaccination. As FHV-1 establishes lifelong latency and reactivates under immunosuppression, and as vaccination attenuates clinical disease but prevents neither latency nor reactivation, these episodes most likely represent recrudescence of latent infection under immunosuppressive therapy rather than a consequence of incomplete vaccination coverage. Diabetes mellitus was recorded in 2/15 PG cats and in none of the CPG cats. Glucocorticoid treatment is a recognized risk factor for diabetes mellitus in cats, but with two events in total these numbers are too small to support any inference about a difference between the cohorts [6,7]. Because adverse events were not assessed by a standardized prospective protocol, they may be underreported, and a drug effect could not always be distinguished from clinical signs of the underlying disease.

Most cats in both cohorts achieved stable disease, and few attained complete remission. Overall mortality was 24.3% (9/37), closely matching the 23.5% previously reported in cats and lower than the 30–52% reported in dogs [2,10,11,12], supporting a comparatively favorable prognosis in cats. Notably, all non-survivors in our cohort were euthanized rather than dying of their disease, so mortality reflects owner decision-making as well as disease severity, and comparisons across studies should be interpreted accordingly. As described in dogs, mortality in our cohort was concentrated early, with eight of nine euthanasia instances performed within the first 18 days and all within four weeks of treatment. This mirrors canine reports in which mortality is highest within the first 14 days, while IMHA-related mortality beyond 90 days falls to approximately 8% and long-term survivors have a favorable prognosis [10,24].

Recurrence of hemolysis was documented in five cats (1/15 PG, 4/22 CPG). Four of these five relapses occurred during ongoing immunosuppressive therapy, on days 159, 184, 557 and 1875, whereas the single PG cat relapsed on day 96, twenty days after complete discontinuation of prednisolone. The numerically higher number of relapses in the CPG cohort is not explained by differences in observation time, as median follow-up was similar in both cohorts (50 vs. 70 days; *p* = 0.70); with one and four events, respectively, this difference does not permit interpretation, and the predominantly short follow-up in both cohorts means that relapse frequency is likely to be underestimated overall. The observed relapse frequency is broadly consistent with reports in cats (up to 31%) and dogs (22.9%), in which recurrence may occur after months to years and is a leading cause of death in animals surviving the acute phase [2,3,10]. That four of five relapses occurred while cats were receiving immunosuppressive therapy, including one cat that developed Evans’ syndrome after five years of continuous CsA treatment, indicates that ongoing immunosuppression does not reliably prevent recurrence and underscores the need for long-term monitoring.

A further observation in our cohort was that very few cats achieved sustained remission after complete discontinuation of immunosuppression: only 2/37 cats (2 of 28 survivors) attained complete remission off therapy, whereas most survivors remained on ongoing immunosuppressive treatment at their most recent follow-up. Both cats discontinued all immunosuppressive medication 820 and 649 days after initiation of treatment and remained relapse-free for a further 4.2 and 1.9 years, respectively. This contrasts with the situation in dogs, in which immunosuppression can often be tapered to complete discontinuation (treatment was discontinued in 33 of 61 dogs in one long-term study, and three months of immunosuppression has been reported to be sufficient to maintain remission in most dogs [10,22]). Our data therefore raise the possibility that cats with non-associative IMHA more frequently require prolonged or lifelong immunosuppression than dogs. This observation should, however, be interpreted with caution, given the small number of complete responders, the retrospective design, the absence of a standardized tapering protocol, and a probable tendency toward more conservative drug withdrawal in a disease less well characterized in cats. This should be regarded as hypothesis-generating and needs prospective evaluation.

The principal limitation of this study is confounding by indication: because CsA was not allocated randomly but was added at the clinician’s discretion in cats judged to be more severely affected at presentation, the two groups differed at baseline, most notably in the severity of anemia at presentation, and no causal effect of CsA can be inferred from these data. All cats in the CPG group began CsA within the first nine days of treatment (median 1.5 days), so treatment allocation reflected the initial assessment of disease severity rather than a documented failure of prednisolone monotherapy. A glucocorticoid-sparing effect of CsA was not assessed, as cumulative prednisolone exposure was not recorded; this hypothesis therefore remains untested.

Additional limitations include the small sample size and retrospective design, the absence of a standardized treatment and tapering protocol with variable prednisolone and CsA dosing, and incomplete long-term follow-up. Housing status (indoor versus indoor–outdoor) was not consistently recorded and could therefore not be reported. Median follow-up was short in both cohorts (50 and 70 days), and approximately one third of cats in each cohort had been lost to follow-up by 90 days. As all deaths occurred within the first four weeks of treatment, this is unlikely to have affected the reported 30- and 90-day mortality. Deaths occurring after referral back to the primary veterinarian may nonetheless have gone unrecorded, so mortality and, in particular, relapse frequency are more likely to be underestimated than overestimated. Because loss to follow-up was almost identical in the two cohorts, this bias is expected to be non-differential.

Cyclosporin A blood concentrations (trough or two-hour post-dose) were not measured, as therapeutic drug monitoring was not routinely performed during the study period. Given the marked inter-individual variability of oral CsA absorption in cats, subtherapeutic exposure in individual cats cannot be excluded and may have attenuated any true treatment effect [25,26,27]. FeLV infection was excluded by antigen ELISA alone. As provirus PCR was not performed, regressive FeLV infection cannot be excluded, and individual cats classified as having non-associative IMHA may have had an undetected underlying retroviral infection [28,29]. Only total reticulocyte counts were available; aggregate reticulocytes, which most accurately reflect current erythropoietic activity in cats, were not reported separately by the laboratory [30]. Adverse events were extracted retrospectively from medical records and may have been underreported. Prospective, randomized studies are needed to establish whether the addition of CsA to prednisolone confers a clinical benefit in cats with non-associative IMHA, a question that cannot be addressed by observational data.

## 5. Conclusions

In this retrospective observational study of 37 cats with non-associative IMHA, most cats presented with profound anemia and an inadequate regenerative response, and most survived the acute phase. Overall mortality was 24.3%, consistent with previous feline reports and lower than the rates described in dogs, supporting a comparatively favorable prognosis in cats; all deaths occurred within the first four weeks, and all were achieved through euthanasia. The majority of surviving cats remained on immunosuppressive therapy at their most recent follow-up, and only two cats achieved sustained remission after complete discontinuation of treatment, lasting 4.2 and 1.9 years. Four of five relapses occurred during ongoing immunosuppressive therapy rather than after its discontinuation, indicating that continued treatment does not reliably prevent recurrence. Together these findings characterize feline non-associative IMHA as a disease that most cats survive but that commonly requires prolonged immunosuppression and long-term monitoring. Because CsA was allocated at the clinician’s discretion and was used preferentially in more severely affected cats, these data do not permit conclusions on the comparative efficacy of the two protocols; prospective, randomized studies are needed to address this question.

## Figures and Tables

**Table 1:** Hematological and biochemical parameters in cats treated with prednisolone alone (PG, *n* = 15) or prednisolone plus cyclosporin A (CPG, *n* = 22). Unless indicated otherwise, values were obtained at presentation, before initiation of immunosuppressive treatment; lowest HCT, WBC, neutrophil and lymphocyte counts refer to the initial hospitalization, and HCT on days 14 and 30 and time to HCT normalization refer to follow-up after initiation of treatment. Values are median [IQR], with the number of cats per parameter; groups were compared using the Mann–Whitney U test.

Parameter	PG (n)	CPG (n)	*p*	RI
HCT at presentation (%)	12.0 [9.2–17.0] (15)	10.2 [7.9–14.3] (22)	0.12	27–47
Lowest HCT (%)	11.0 [9.4–16.5] (15)	8.5 [7.1–12.4] (22)	0.06	27–47
HCT day 14 (%)	22.7 [18.0–26.8] (9)	20.5 [15.2–24.1] (16)	0.47	-
HCT day 30 (%)	28.7 [23.5–37.8] (8)	27.3 [24.6–33.5] (12)	0.75	-
Time to HCT normalization (days)	22.0 [15.5–58.0] (9)	34.5 [13.5–48.0] (10)	0.84	-
Hemoglobin at presentation (g/dL)	5.1 [3.4–6.2] (15)	3.6 [2.5–5.2] (22)	0.03	8–17
Reticulocytes (/µL)	36,417 [17,608–60,860] (15)	30,736 [11,859–83,196] (22)	0.57	>60,000
Platelets (/µL)	118,000 [43,000–159,000] (15)	112,000 [27,250–240,000] (22)	0.99	180,000–430,000
Lowest WBC (/µL)	7182 [5060–9240] (15)	6035 [4223–7686] (22)	0.19	6000–18,000
Highest WBC (/µL)	13,050 [7270–16,470] (15)	12,985 [9460–17,840] (22)	0.35	6000–18,000
Lowest neutrophils (/µL)	5004 [3000–6003] (15)	3230 [2174–4751] (22)	0.06	3600–12,750
Highest neutrophils (/µL)	8066 [4698–12,183] (15)	7461 [3861–11,711] (22)	0.77	3600–12,750
Lowest lymphocytes (/µL)	1866 [670–3114] (15)	868 [472–1709] (22)	0.19	900–5100
Total bilirubin (mg/dL)	0.83 [0.17–1.97] (14)	0.43 [0.23–1.71] (21)	0.72	<0.2
ALT (U/L)	106 [40–175] (15)	116 [44–198] (22)	0.69	<100
ALKP (U/L)	31 [17–49] (13)	26 [17–37] (22)	0.68	<30
Urea (mg/dL)	39.4 [23.5–49.2] (14)	43.7 [33.6–55.8] (20)	0.29	20–65
Creatinine (mg/dL)	1.1 [0.8–1.4] (15)	1.0 [0.9–1.3] (21)	0.80	<1.6
Serum amyloid A (mg/L)	35.4 [6.9–99.0] (9)	10.7 [4.4–18.0] (12)	0.09	<5

ALKP, alkaline phosphatase; ALT, alanine aminotransferase; HCT, hematocrit; IQR, interquartile range; SAA, serum amyloid A; WBC, white blood cells; RI, reference interval.

**Table 2:** Adverse events recorded during treatment, by group (number of cats affected).

Adverse Event	PG (n = 15)	CPG (n = 22)
Polyphagia	2	5
Polyuria/polydipsia	1	2
Acute kidney injury	0	1
Hyporexia/inappetence	3	1
Bacterial cystitis	0	0
Excessive hair loss	0	2
Lethargy	2	5
Vomiting/nausea	2	1
Feline upper respiratory tract disease	0	3
Congestive heart failure	0	1
Diabetes mellitus	2	0
Pica	1	1
Diarrhea	2	1

**Table 3:** Outcome and mortality by group.

Outcome	PG (n = 15)	CPG (n = 22)	*p*
Complete remission	1 (6.7%)	1 (4.5%)	>0.99
Stable disease	10 (66.7%)	16 (72.7%)	0.73
Euthanasia	4 (26.7%)	5 (22.7%)	>0.99

Groups were compared using Fisher’s exact test. All deaths were euthanasia performed within the first four weeks of treatment; 30-day and 90-day mortality were therefore identical.

## Data Availability

The data presented in this study are available on request from the corresponding author due to privacy restrictions applying to clinical records of client-owned animals and their owners.

## Abbreviations

The following abbreviations are used in this manuscript:

ACVIM	American College of Veterinary Internal Medicine
ALKP	Alkaline phosphatase
ALT	Alanine aminotransferase
CPG	Cyclosporin A—prednisolone group
CRP	C-reactive protein
CsA	Cyclosporin A
DAT	Direct antiglobulin test (=Coombs test)
ELISA	Enzyme-linked immunosorbent assay
FeLV	Feline leukemia virus
FIV	Feline immunodeficiency virus
Hb	Hemoglobin
HCT	Hematocrit
IMHA	Immune-mediated hemolytic anemia
IQR	Interquartile range
PCR	Polymerase chain reaction
PG	Prednisolone group
RCP	Rhinotracheitis, calicivirus and panleukopenia
SAA	Serum amyloid A
WBC	White blood cell
